# Hsp60 response in experimental and human temporal lobe epilepsy

**DOI:** 10.1038/srep09434

**Published:** 2015-03-24

**Authors:** Antonella Marino Gammazza, Roberto Colangeli, Gergely Orban, Massimo Pierucci, Giancarlo Di Gennaro, Margherita Lo Bello, Alfredo D'Aniello, Fabio Bucchieri, Cristoforo Pomara, Mario Valentino, Richard Muscat, Arcangelo Benigno, Giovanni Zummo, Everly Conway de Macario, Francesco Cappello, Giuseppe Di Giovanni, Alberto J. L. Macario

**Affiliations:** 1 Department of Experimental Biomedicine and Clinical Neurosciences, University of Palermo, Palermo, Italy; 2 Euro-Mediterranean Institute of Science and Technology, Palermo, Italy; 3 Department of Physiology and Biochemistry, Faculty of Medicine and Surgery, University of Malta, Msida, Malta; 4 NEUROMED, IRCCS, Pozzilli, Italy; 5 Department of Anatomy, Faculty of Medicine and Surgery, University of Malta, Msida, Malta; 6 Department of Forensic Pathology, University of Foggia, Foggia, Italy; 7 Department of Microbiology and Immunology, School of Medicine, University of Maryland at Baltimore; and IMET, Columbus Center, Baltimore, MD, USA; 8 Institute “Paolo Sotgiu” for Research in Quantitative and Quantum Psychiatry and Cardiology, University of Human Sciences and Technology (LUDES), Lugano, Switzerland; 9 Neuroscience Division, School of Bioscience, Cardiff University, Cardiff, UK

## Abstract

The mitochondrial chaperonin Hsp60 is a ubiquitous molecule with multiple roles, constitutively expressed and inducible by oxidative stress. In the brain, Hsp60 is widely distributed and has been implicated in neurological disorders, including epilepsy. A role for mitochondria and oxidative stress has been proposed in epileptogenesis of temporal lobe epilepsy (TLE). Here, we investigated the involvement of Hsp60 in TLE using animal and human samples. Hsp60 immunoreactivity in the hippocampus, measured by Western blotting and immunohistochemistry, was increased in a rat model of TLE. Hsp60 was also increased in the hippocampal dentate gyrus neurons somata and neuropil and hippocampus proper (CA3, CA1) of the epileptic rats. We also determined the circulating levels of Hsp60 in epileptic animals and TLE patients using ELISA. The epileptic rats showed circulating levels of Hsp60 higher than controls. Likewise, plasma post-seizure Hsp60 levels in patients were higher than before the seizure and those of controls. These results demonstrate that Hsp60 is increased in both animals and patients with TLE in affected tissues, and in plasma in response to epileptic seizures, and point to it as biomarker of hippocampal stress potentially useful for diagnosis and patient management.

Epilepsy is one of the most common chronic neurologic disorders affecting approximately 1% of the world population^1^,^2^. This disease has deleterious effects on the quality of life affecting independent living, education, employment, mobility, and personal relationships. Epilepsy is characterized by spontaneous recurrent seizures (SRSs) caused by abnormal, synchronized, high frequency neuronal discharges^3^. Neuronal excitability can be affected by mitochondrial alterations such as depletion of ATP, generation of ROS, elevated oxidative stress, disruption of Ca^2+^ homeostasis, dysregulation of excitotoxicity, and alterations in biosynthesis and metabolism of neurotransmitters^4^.

The most common type of epilepsy in adult humans is temporal lobe epilepsy (TLE), characterized by a progressive development of SRSs from temporal lobe foci and unique morphological alteration in the hippocampus^2^,^5^. Usually, TLE is initiated by a first hit, such as head trauma or stroke, brain infection, or febrile seizures that induce a status epilepticus (SE). The period between the initial injury and the occurrence of the first epileptic seizure is named epileptogenesis. This is a clinically silent period of 5–10 years in which a cascade of neurobiological events, and histological and biochemical changes occur^3^. The chronic process that follows the initial insult involves neuronal activation with intracellular calcium accumulation, and activation of gene expression and protein synthesis. Inflammation develops at the site of injury, involving glial and endothelial cells^6^. At the later stage of epileptogenesis, sprouting of new axons and synapses, and angiogenesis occur changing the nerve tissue microarchitecture^7^. Recently, experimental and clinical data have also demonstrated a causative role played by inflammation in TLE comorbidities such us mood disorders and cognitive impairments^8^. Therefore, biochemical measurements of inflammatory mediators in blood and serum might reflect the degree and extent of brain inflammation and, thus, provide powerful tools for diagnostic, prognostic and therapeutic purposes for example in TLE due to hippocampal sclerosis patients^8^.

Among the neuroinflammatory mediators, heat shock proteins (Hsps) can be considered promising candidates as useful biomarkers in CNS disorders including TLE^9^. Many Hsps are molecular chaperones constitutively expressed under normal temperature that have indispensable functions in the life cycle of proteins, and play a role in protecting cells from deleterious stressors. Molecular chaperones are able to inhibit the aggregation of partially denatured proteins and refold them in order to maintain protein homeostasis and tissue physiology. In this regard, these proteins offer a promising alternative for protection against stressors, and as therapeutic agents for diseases caused by protein misfolding^10^,^11^. Numerous Hsps are critical regulators in normal neural physiology as well as in cell stress responses^12^,^13^. Emerging evidence involves Hsps in synaptic transmission, autophagy, stress response related to the endoplasmic reticulum, protein kinase and cell death signaling. These functions, together with the classical functions of molecular chaperones, indicate that manipulation of Hsps may have significant effects on the fate of cells in neurological injury and disease states^13^.

One member of the Hsps is Hsp60, a mitochondrial protein constitutively expressed under normal conditions and induced by different types of stressors such as heat shock, oxidative stress, and DNA damage^14^. Inside mitochondria, Hsp60 together with Hsp10 constitute a folding machine for the correct folding of other mitochondrial proteins^15^,^16^. In the brain, Hsp60 is endogenously expressed in astrocytes, neurons, microglia, oligodendrocytes, and ependymal cells^17^. This distribution suggests an active participation of this chaperonin in many functions of the brain both in normal and pathological conditions.

Recently, new locations and functions have been found for this chaperonin, also related to its structural characteristics, describing Hsp60 as an ubiquitous molecule with multiple roles in health and disease^18^,^19^. Hsp60 can accumulate in the cytosol and plasma membrane, reaching the extracellular space via secretory vesicles^18^,^20^. In the extracellular environment, Hsp60 can interact with receptors present on immune cells^21^,^22^ and reach the bloodstream acting as a “chaperokine” at distant sites^23^. Hsp60 participates in a number of inflammatory and autoimmune processes^24^,^25^, including some affecting the nervous system, such as multiple sclerosis and myasthenia gravis^10^,^26^. In view of the information available about the various roles of Hsp60 in the central nervous system, in the present study we firstly investigated the expression and distribution of Hsp60 in the rat hippocampus. We used a model of partial complex (limbic) seizures, based on the phenomenon of maximal dentate activation (MDA) recorded in the dentate gyrus (DG), which is induced by repetitive electrical stimulation of the perforant path (PP) in anesthetized rats^27^,^28^,^29^. The analysis was conducted using Western blotting and immunohistochemistry on hippocampal tissue samples. The levels of Hsp60 in the blood of epileptic rats were determined using ELISA. The same technique was used to assay the levels of Hsp60 in the blood of patients before and after the occurrence of a temporal lobe seizure.

## Results

### Maximal dentate activation (MDA)

**Figure 1:**
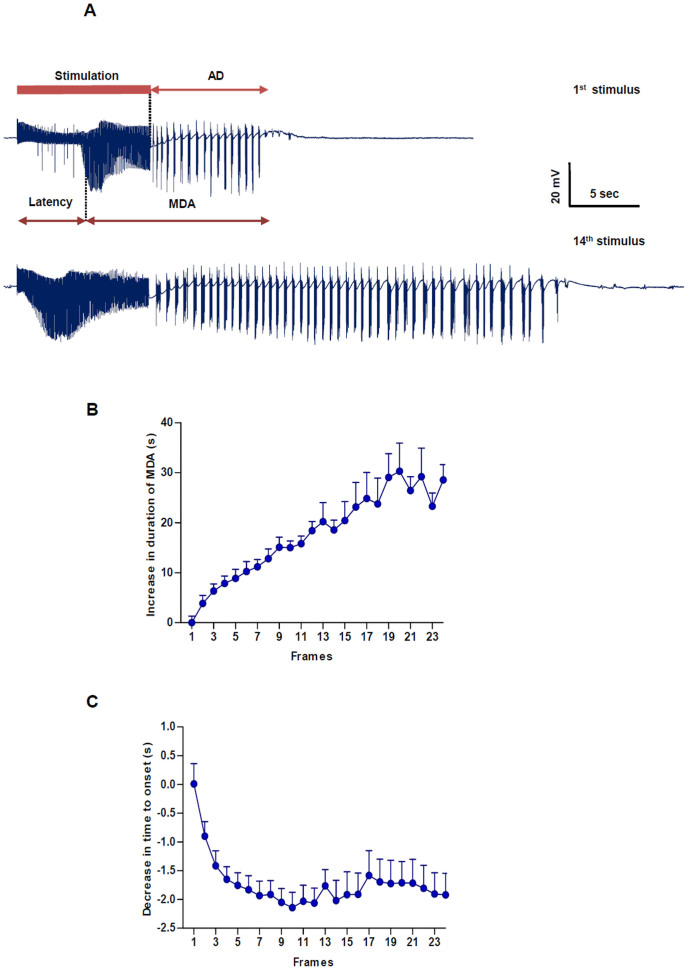
Maximal dentate activation (MDA). Representative electrophysiological recording of MDA and after discharge (AD) recorded in alternating current (AC) mode in the dentate gyrus of the hippocampus induced by 10 s electrical stimulation at 20 Hz in a urethane-anesthetized rat. (A) The Time to onset is defined by the time that occurs from the beginning of the stimulus train to the midpoint of the maximum amplitude of the PSs. Note the increase in MDA and AD between the first and the 14^th^ stimulus. (B) Duration and (C) time to onset of MDA, which were measured for each stimulus train. These values were then normalized, averaged and plotted (± SEM) against stimulus number. Each solid square represents the mean value from 9 animals.

MDA was electrophysiologically characterized by (1) the onset of large-amplitude (*i.e*., 20–40 mV) population spikes (PSs) fired in bursts, and (2) a rapid increase in amplitude of DG PS^28^,^29^. Stimulus trains of 10 s (pulses of 0.3 ms duration, at 20 Hz) were delivered through the PP electrode at an initial intensity of 200 μA. If MDA was not elicited, the stimulus intensity was increased in steps of 50 μA and redelivered every 2.5 min until MDA was induced. Usually, threshold was reached at 350 ± 100 μA, and stimulus intensity was further increased by 100 μA. For each stimulus, the duration of MDA, time to onset and after discharge (AD) were measured as shown in Fig. 1A. Time to onset and duration of MDA were measured as repeated seizure-inducing trains and were delivered every 10 min for the next 4 h (total of 24 stimulus trains). As shown in Fig. 1A, the latency to MDA onset was measured from stimulus onset to the point of PS appearance with half of the maximal amplitude. The duration of MDA increased and the time to onset gradually decreased. The measured durations of MDA and time to onset (Fig. 1B) were ‘normalized' by subtracting their duration in response to the first stimulus from the duration in response to each subsequent stimulus train. Thus, for individual stimulus trains after the first, a change in duration (or time to onset) was calculated. In this way, data from separate animals were averaged. There was a gradual increase in the change of duration of MDA over the first 14 stimuli (Fig. 1A,B) which then became quite variable reaching 24.5 ± 2.4 s (n = 9) after the 24^th^ stimulus. There was also a gradual decrease in the time of onset to −2.2 ± 0.3 s (at the 8^th^ stimulus) and then a plateau (Fig. 1A,C).

### Effect of MDA on Hsp60 levels in the hippocampus measured by Western blotting

**Figure 2:**
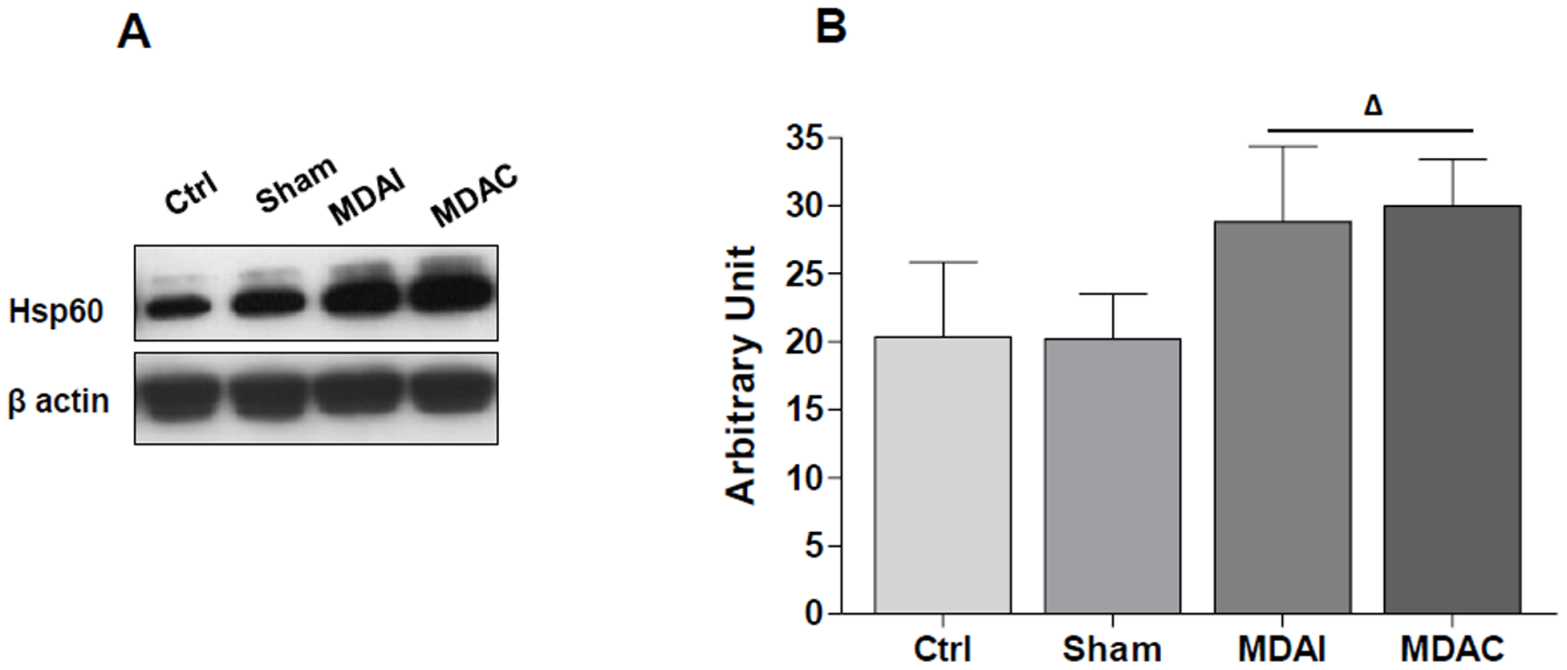
Hsp60 levels in the hippocampus of control and MDA-stimulated rats. (A) Representative cropped blots for Hsp60 in control (Ctrl), without stimulation (sham), right ipsilateral (MDAI) and in contralateral (MDAC) hippocampus to the stimulation of the perforant pathway (PP). The gels were run under the same experimental conditions and β-actin was used as an internal control. (B) Ratio Hsp60 levels/β-actin levels as a reflection of Hsp60 increase (mean ± SD). One-way ANOVA for repeated measurements followed by Bonferroni post-hoc test; ^Δ^p ≤ 0.05 vs. Ctrl and Sham.

Hsp60 was quantified in lysates of the hippocampi, derived from control rats (Ctrl, n = 6), rats with the electrodes positioned in hippocampus and left in place for a total of 4 h without stimulation (shams, n = 5) and MDA-stimulated rats (n = 9) (Fig. 2A,B). Hsp60 levels were normalized to beta actin levels. Hsp60 was increased up to 1.5 fold of the basal level for MDAI (right hippocampus ipsilateral to the PP stimulation) and for MDAC (left hippocampus contralateral to the PP stimulation) (p ≤ 0.05 vs Ctrl and sham animals) (Fig. 2B). The difference between Ctrl and sham animals as well as the difference between MDAI and MDAC were not statistically significant (Fig. 2A,B).

### Hsp60 levels in the dentate gyrus and hippocampus proper following MDA

**Figure 3:**
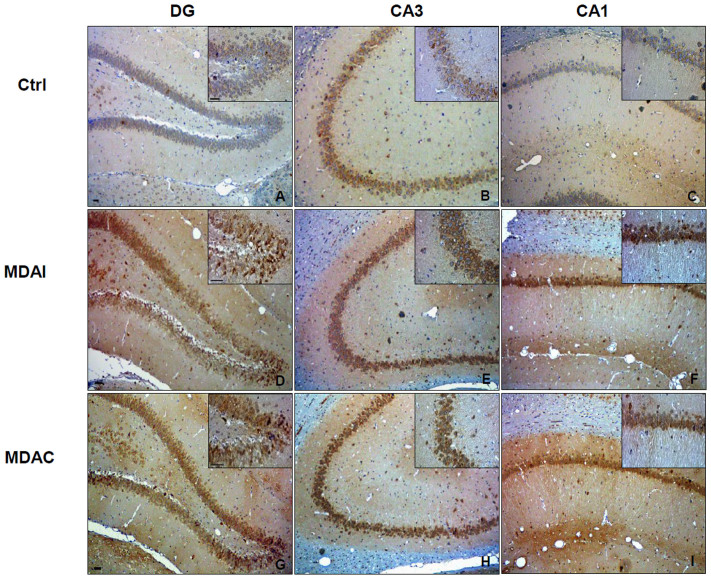
Levels and preferential localization of Hsp60 in control non-stimulated rats (Ctrls) and in MDA-stimulated rats. (A–C) Immunohistochemical staining for Hsp60 in the hippocampus of control non-stimulated rats. (D–F) Representative microphotographs of Hsp60 levels in the hippocampus of MDA-stimulated rats, ipsilateral to the perforant pathway (PP) stimulation (MDAI). (G–I) Representative microphotographs of Hsp60 levels in the hippocampus of MDA-stimulated rats, contralateral to the PP stimulation (MDAC). As shown in the microphotographs (A–C) and in the relative insets, in Ctrl animals Hsp60 was detected in all the hippocampal sectors. In the dentate gyrus (DG), Hsp60 immunoreactivity was in the granular cell layer while in the CA3 sector and CA1 sector was for the most part in the pyramidal cell layer. The illustrative images of MDA-stimulated rats (D–I) and the relative insets show that Hsp60 immunoreactivity was increased in the DG, CA3 and CA1 sectors both in MDAI and MDAC compared to Ctrl rats. This Hsp60 increase was observed on neuron somata and neuropil. Bar = 100 μm.

**Figure 4:**
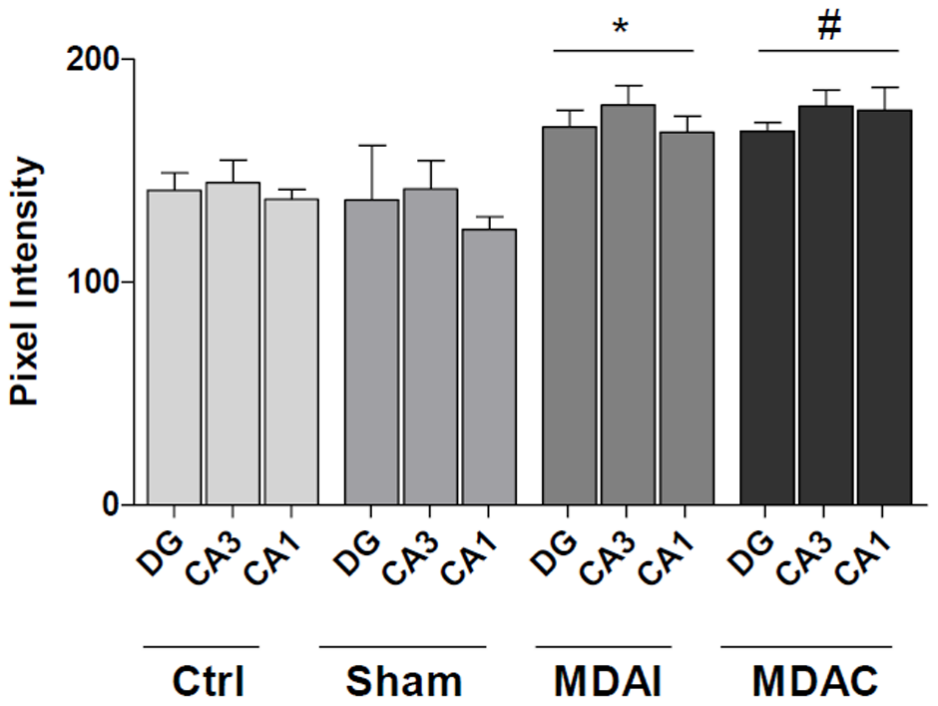
Increase in Hsp60 levels in MDA-stimulated rats. Representative histograms showing densitometric measurements of the Hsp60 staining intensity in controls, shams, and MDA-stimulated rats. Staining intensity was expressed as pixel intensity. Ctrl: Control; Sham: Animals with the electrodes left in place for the entire experiment without stimulation; DG: Dentate gyrus; MDAI: ipsilateral to MDA-stimulation; MDAC: controlateral to MDA-stimulation. *^#^p ≤ 0.001 vs Ctrl and sham.

To determine the localization of Hsp60, immunostaining was performed. In Ctrl animals (Fig. 3) as well as in sham animals (not shown) Hsp60 was detected in all the hippocampal sectors such as DG, CA3 and CA1 (Fig. 3A–C). Hsp60 immunoreactivity was localized in the DG granule cells (Fig. 3A), and in CA3 (Fig. 3B) and CA1 pyramidal cells (Fig. 3C). There was also a diffuse neuropilar labeling of undetermined association with specific elements. In MDA-stimulated rats Hsp60 immunoreactivity was increased in the DG (Fig. 3D,G), CA3 (Fig. 3E,H), and CA1 (Fig. 3F,I), both in MDAI and MDAC compared to Ctrl rats, along the strata of these hippocampal sectors. This Hsp60 increase was observed on neuron somata and neuropil. The Hsp60 immunoreactivity increase in MDA-rats compered to Ctrl and sham rats was statistically significant (Fig 4, p ≤ 0.001).

### Hsp60 levels in the plasma of MDA-stimulated rats

**Figure 5:**
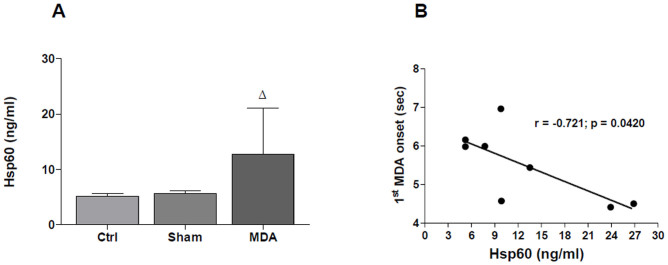
Increase in Hsp60 plasma levels in MDA-stimulated rats. (A) The levels of Hsp60 in plasma of MDA-stimulated rats are elevated significantly in comparison with rats without stimulation (sham) and with the controls (Ctrl). ^Δ^p ≤ 0.05 vs. Ctrl and sham. Ctrl: Control. (B) Plasma levels of Hsp60 negatively correlate with the onset of the first maximal dentate activation in a model of temporal lobe epilepsy in rats. Pearson correlation analysis was performed to describe relationships between the onset of the first maximal dentate activation induced by electrical activation of the perforant path (stimulus duration 0.3 ms, 20 Hz, per 10 sec) and the plasma Hsp60 levels measured at the end of the experiment (24 stimulations every 10 min). An alpha level of 0.05 was chosen as statistical threshold. The time to onset of the first MDA correlated negatively with serum Hsp60 levels (r = −0.721, p = 0.0420). The time to onset is defined as time that occurs from the beginning of the stimulus train to the midpoint of the maximal amplitude of the spike population.

The difference between plasmatic levels of Hsp60 in MDA-stimulated rats compared to the plasmatic levels in shams (p ≤ 0.05) and Ctrls (p ≤ 0.05) was statistically significant. No significant differences between Hsp60 plasmatic levels in sham rats compared to the Ctrls were revealed. Hsp60 levels in MDA-stimulated rats (n = 9) ranged between 5.23 ng/ml and 26.88 ng/ml (mean: 12.76 ± 8.28 ng/ml) and in sham rats (n = 5) ranged between 3.95 ng/ml and 9.79 ng/ml (mean: 6.52 ± 2.51 ng/ml). Hsp60 plasmatic levels in Ctrl rats (n = 6) ranged between 3.40 ng/ml and 6.76 ng/ml (mean: 5.11 ± 1.36 ng/ml) (Fig. 5A). Moreover, there was a negative correlation between the onset of the first MDA induced by electrical activation of the PP and the plasma Hsp60 levels measured at the end of the experiment (Pearson's r = −0.721, p ≤ 0.05) (Fig. 5B). No other correlations were revealed between Hsp60 and MDA parameters (not shown).

### Hsp60 levels in the plasma of subjects with epilepsy

**Figure 6:**
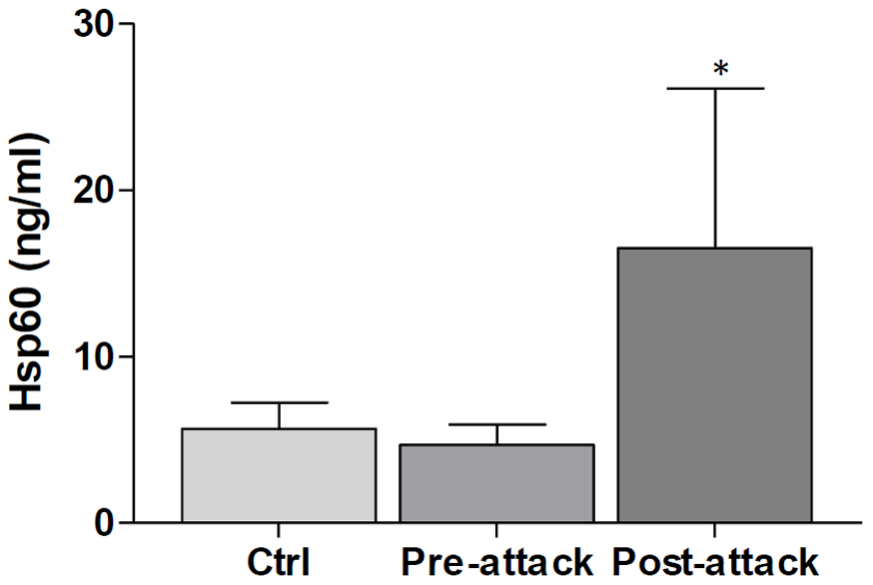
Increase in Hsp60 plasma levels in patients after epileptic seizure. The levels of Hsp60 in plasma after the seizure were elevated significantly in comparison with the pre-seizure levels, which were the same as those in normal controls. *p ≤ 0.05 vs controls and pre-attack group. Ctrl: Control.

The difference between plasmatic levels of Hsp60 in patients after epileptic seizure (n = 10), compared to levels in the same subjects before the epileptic seizure was statistically significant (p ≤ 0.05), as well as when it was compared to Ctrls (p ≤ 0.05). Hsp60 levels ranged between 3.22 ng/ml and 6.41 ng/ml (mean: 4.74 ± 1.22 ng/ml) in the plasma of patients before the attack and between 5.72 ng/ml and 31.72 ng/ml (mean: 11.81 ± 5.73 ng/ml) in plasma of patients after the attack. By contrast, there was no difference between Hsp60 plasmatic levels in patients before the attack and levels in Ctrl subjects. Hsp60 plasmatic levels in Ctrl subjects (n = 10) ranged between 4.01 ng/ml and 9.39 ng/ml (mean: 5.34 ± 2.03 ng/ml) (Fig. 6).

## Discussion

To fully appreciate the physiopathological and clinical (practical) significance of the observations reported, the following topics, from the general to the specific, must be discussed: (i) the impact of stress on Hsps in the CNS; (ii) the association between Hsp60 in the CNS and TLE; (iii) the association of TLE and Hsp60 in mitochondria with oxidative stress; and (iv) the association of Hsp60 beyond mitochondria with TLE.

The impact of stress on Hsps in the CNS has been examined for years, and it has been suggested that stress induces elevation of Hsps as a mechanism of cellular defense to injury^10^. However, existing data also suggest the involvement of Hsps in neuronal damage caused by SE, although their role in neurodegeneration during epilepsy still remains uncertain^9^,^30^,^31^,^32^,^33^. In animal models of epilepsy, increased Hsp70 expression during acute^34^ and chronic phases^35^ has been documented. In TLE patients, complete remission of mesial TLE seizures postsurgery was associated with decreased Hsp70 expression in CA4 and subiculum and decreased Hsp90 expression in the granular layer^36^. Higher Hsp70 serum levels in patients with TLE as compared to controls were observed, and were predictive of higher frequencies of seizures in the TLE group^9^. Data from animal models showed that Hsp72 increased levels in specific hippocampal neuronal subpopulations correlate with limbic seizure intensity and duration^37^ and Hsp27 was found to be a highly sensitive and specific hippocampal marker for full development of pilocarpine-induced SE^38^.

In contrast to other Hsps, Hsp60 levels and expression have only been sporadically studied in animal models of CNS diseases or neurological patients. Hsp60 was found increased in the brain stem after subarachnoid hemorrhage, forebrain or focal cerebral ischemia, and neonatal hypoxia-ischemia in rats^13^. Hsp60 was found in the protein aggregates typical of neurodegenerative diseases such as Parkinson's disease (PD) and Alzheimer's disese (AD), in which a fundamental role is played by oxidative stress and mitochondrial dysfunction^39^,^40^. Physiologically, neural expression of Hsp60 increases over the course of development, consistent with the changes of mitochondrial content in the brain^17^.

The association between Hsp60 in the CNS and TLE, the most common type of epilepsy in humans, is poorly understood, and our study is the first aiming at elucidating this important issue. We investigated tissue levels and distribution of Hsp60 in the MDA animal model of TLE. This is an electrographic kindling model using urethane-anaesthetized rats, marker for reverberatory seizure activity in hippocampal-parahippocampal circuits^28^,^29^,^30^. The MDA model has been extensively used to study the phenomenon of epileptogenesis and the effects and mechanisms of new anticonvulsant drugs^28^,^29^,^30^. In addition, we measured Hsp60 in the blood of epileptic rats, and in the plasma of patients before and after temporal lobe seizures. Immunohistochemistry and Western blotting analyses showed that the levels of Hsp60 were significantly increased in hippocampal neurons of MDA rats when compared to the controls, including sham treated rats. Surprisingly, a similar increase occurred in both ipsilateral and contralateral hippocampus to the PP stimulation. These data are in contrast with the observation of a decrease in Hsp60 levels, assayed by proteomic analysis, in the rat hippocampus after pilocarpine treatment^5^. This difference may be explained because while we used acute electric kindling (*i.e.*, the MDA model) the other investigators used chemical stimulation (*i.e*., the pilocarpine model). Moreover, the timing of the collection of the brains was different, after 4 h in our case instead of 12 and 72 h after the pilocarpine-induced SE experiments^5^.

Any investigation of the possible role of Hsp60 in any disease must consider also mitochondria, the canonical residence of the chaperonin, and oxidative stress, typically affecting that organelle. Therefore, the possible association of TLE and Hsp60 in mitochondria with oxidative stress must be discussed in this report. TLEs are a group of acquired neurological disorders in which the humans and animals affected experience recurrent epileptic seizures arising from one or both temporal lobes of the brain^41^,^42^. The large quantity of mitochondria present in the brain makes this tissue particularly vulnerable to oxidative stress-induced damage with high degree of oxygen consumption and less antioxidant capacity. Mitochondrial oxidative stress and dysfunction have been suggested to be contributing factors to the development of neurological disorders, TLE in particular, but their precise role (cause or consequence) in epileptic seizures has not yet been fully characterized^41^. Mitochondrial dysfunction and oxidative stress are factors that not only occur acutely as a result of precipitating injuries such as SE, but may also contribute to neuronal cell death, epileptogenesis, and chronic epilepsy. Therefore, Hsp60, a constitutive mitochondrial protein with specific functions related to mitochondrial protein folding, especially in response to oxidative stress^43^, might play an important role in the physiopathology of this type of epilepsy. Overexpression of Hsp60 was associated with increased activity of mitochondrial complex I after 3,4-dihydroxy-L-phenylalanine (L-DOPA) administration to rats^39^. Hsp60 can be induced by mitochondrial DNA depletion^14^ and this chaperonin can interact directly with other mitochondrial proteins such as aldehyde dehydrogenase 2, ATP synthase, dihydrofolate reductase, and human carbonicanhydrase II^11^. Likewise, Hsp60 associates with pro-caspase 3 favoring cell survival^44^ and some of these Hsp60 interactors are affected by oxidative stress leading to the metabolic alterations that characterize TLE^45^. Hsp60 induction could be considered as a protective mechanism against epileptic seizures as supported by the observation that a loss of function of Hsp60 leads to an increased vulnerability to oxidative stress^5^, which in turn can affect neuronal excitability and seizure susceptibility^41^.

It is also pertinent to discuss our findings considering the association of Hsp60 beyond mitochondria with TLE because over the last few years it has become clear that Hsp60 not only resides and works in the mitochondria but also elsewhere, in the cytosol, plasma cell membrane and outside the cell, including the blood stream^20^,^25^,^46^. For instance, Hsp60 was detected in the culture supernatants in a cellular Parkinson's disease model^47^ and in sera of patients with brain tumor, in which the chaperonin was secreted via exosomes that escaped the brain-blood barrier (BBB) and, thus, was endowed with a potential systemic and distal signaling property^48^. Since seizures can induce brain inflammation and BBB breakdown^49^,^50^ in both epileptic rats and humans^51^, we can hypothesize that a similar scenario leads to appearance of Hsp60 in the blood in TLE. The release from neural cells might occur in soluble form, possibly via Golgi or through microvesicles, e.g., exosomes, but this is a field open to investigation. In our experiments, Hsp60 significantly increased in the plasma of MDA stimulated rats and in the plasma of patients after epileptic seizure. Moreover, we observed a negative correlation between the onset of the first MDA induced by electrical activation of the PP and Hsp60 release in the circulation in the animal model of TLE. It has been suggested that the latency to onset of MDA can be used as a gauge of seizure threshold^27^; this finding suggests that the animals with intrinsic higher hippocampal excitability have higher levels of plasma Hsp60 after induction of MDA, revealing a possible role for Hsp60 in the susceptibility to TLE. On the other hand, Hsp60 release induced by MDAs might be a protective strategy from oxidative stress as it occurs for other neurodegenerative diseases such as AD^52^. Seizures induce ROS generation, steady-state levels of ROS increase and oxidative damage arises when the antioxidant system and repair processes fails. A vicious cycle occurs including chronic seizures that generate ROS, which increase above manageable levels: the whole process leading to chronic epilepsy^41^. Therefore, elevated Hsp60 in plasma may be considered a stress biomarker, reflecting neuronal cell damage in the hippocampus, similarly to serum levels of Hsp70^9^. Nevertheless, we did not observe a positive correlation between the amount of expression of hippocampal Hsp60 and its plasma levels in MDA rats and, therefore, sources of plasma Hsp60 other than the affected hippocampus cannot be excluded.

The preceding discussion of our data within the context of information from other laboratories, unveils a cause-effect line of associations in time (with reference to seizures) and space (cells and tissues) between stress, oxidative stress in particular, mitochondrial involvement, and Hsp60 in the mitochondria and beyond the organelle in the pathogenesis of TLE. While the intimate molecular mechanisms of these associations and of the way they directly cause signs and symptoms in TLE have still to be elucidated, the data reported here are useful in at least two directions: a) Hsp60 levels in affected tissues and the patients' blood are clearly altered in relation to seizures. Therefore, it is justified to launch investigations on the intimate molecular mechanisms of the chaperonin's participation in the initiation of epileptogenesis and disease progression and, by extension, to develop treatments using the chaperonin as therapeutic target or agent, depending on whether it is found pathogenic or cytoprotective, respectively; and b) Hsp60 in blood is a seemingly reliable diagnostic biomarker. Hence, its clinical practical utility to follow patient status, including response to treatment, should be assessed routinely in the evaluation of humans suffering TLE.

## Methods

### Animal Studies

#### Maximal dentate activation

Experiments on animals were performed at the Department of Physiology and Biochemistry Faculty of Medicine and Surgery, University of Malta, Msida, Malta. The care and treatment of all animals were carried out in accordance with the EU Council Directive 86/609/EEC, the Animals Scientific Procedures Act 1986. All experimental protocols were approved by the Faculty of Medicine and Surgery Animal Care and Use Committee, University of Malta. Every necessary effort was made to minimize the animals' pain and suffering and to reduce the number of animals used. Experiments were conducted on male Sprague–Dawley rats weighing ~300 g from the Charles River Laboratories (Calco, Lecco, Italy). The rats were housed under a 12 h light-dark cycle (lights on at 7:00 a.m.) at a constant temperature (21 ± 1°C) and relative humidity (60 ± 5%).

Rats were anesthetized by i.p. urethane (Sigma-Aldrich, Dorset, England) (1.2 g/kg) administration and positioned in a David Kopf stereotaxic frame. Body temperature was maintained by a heating pad and a temperature controller unit (Temperature Control Unit HB 101/2, Letica Scientific Instruments, Laguna Niguel, CA). Field potentials were evoked by stimulating the PP (AP: −8.3 L: 4.8 V: 3.4)^53^ with a bipolar stimulating electrode (bifilar twisted stainless steel, Stainless Steel Wire AISI316, Advent). The recording electrode (Tungsten Low Resistance Electrode, A-M Systems Inc., Carlsborg, WA) was implanted into the hilus of the DG of the hippocampus (AP: −4.2 L: 2.2 V: 3.6)^53^. During the surgical procedure, electrodes were advanced slowly downward until reaching the optimal depth to record PSs. In order to record the electrical field during implantation and experiment, a NeuroLog amplifier (Digitimer Ltd, high pass: 0.2 Hz, low pass: 5,000 Hz, gain: 200) was used. Square-wave pulses of 0.2 ms duration were applied at 1 per minute, using a constant current stimulator (Digitimer Ltd, model DS3) and a digitally controlled stimulator. After the optimal depth for recording PS had been reached, control data were acquired after a 60 min delay to allow the tissue to recover from any trauma due to electrode implantation. Stimulus intensity was set to evoke 40–50% of the maximum amplitude of the PS. Recording of PS was performed using the same settings as during implantation (see above). Responses were digitized by a CED 1401 plus analogue–digital converter (Cambridge Electronic Design Ltd., Cambridge, UK), stored on a computer and averaged offline using Signal 1.9 software. Sampling rate was set to 10 kHz. Location of the recording electrode was verified histologically. The same recording and stimulation procedure were used under sham implantation. Sham animals had electrodes positioned in the PP and DG, respectively. The electrodes were than left in place for a total of 4 h without stimulation.

### Tissue collection

For immunohistochemistry, Ctrl rats, shams, and MDA-stimulated rats (n = 9 per group) were transcardially perfused with 0.01 M phosphate buffer saline (PBS, pH 7.4), and then with chilled 4% paraformaldehyde. After post-fixation, the brains were embedded in paraffin and sectioned coronally (5 μm) using a microtome. For Western blot analysis the hippocampus of each rat was dissected, homogenized in cold RIPA buffer (0.3 M NaCl, 0.1% SDS, 25 mM HEPES pH 7.5, 1.5 mM MgCl2, 0.2 mM EDTA, 1% Triton X-100, 0.5 mM DTT, 0.5% sodium deoxycholate) containing protease inhibitor cocktail (Sigma Aldrich) and stored at −20°C until use. For ELISA tests whole blood samples were collected from Ctrls (n = 6), shams (n = 5), and MDA-stimulated rats (n = 9) in EDTA-treated tubes and centrifuged for 15 min at 1000 × g at 4°C within 30 min of collection. The plasma was removed, and stored in aliquot at −80°C until use.

### Western blotting

Western blotting was performed as previously described^54^. Equal amounts of proteins (40 μg) were separated on SDS-PAGE and transferred onto a nitrocellulose membrane (BioRad, Segrate, Italy). After blocking with 5% albumin bovine serum (Sigma Aldrich), membranes were probed with primary antibodies (mouse anti-Hsp60 monoclonal antibody, and mouse anti-β actin monoclonal antibody, Sigma Aldrich, Milan, Italy) diluted at 1:1,000 overnight at 4°C. Protein bands were visualized using the enhanced chemiluminescence (ECL) detection system (GE Healthcare Life Sciences, Milan, Italy), and the data were evaluated and quantified using ImageJ Free software (NIH, Bethesda, MD). Each experiment was performed at least three times.

### Immunohistochemistry

Brain sections involving the hippocampus were mounted on tissue slides and deparaffinized. Sections were immersed in 0.3% H_2_O_2_ for 5 min to quench endogenous peroxidase activity, and treated with 10 mM, pH 6.0, 0.05% Tween 20 tri-sodium citrate at 95°C for 8 min for antigen retrieval. Subsequently, immunohistochemistry was performed using the Histostain-Pluss IHC detection Kit (Histostain-plus Kit^3rd^ Gen IHC Detection Kit, Life Technologies, Monza, Italy) and a primary antibody against human Hsp60 (mouse anti-Hsp60 monoclonal antibody, Sigma Aldrich, dilution 1:400). Appropriate positive and negative controls, were run concurrently (Fig. S1).

Nuclear counterstaining was done using hematoxylin (DAKO, Carpinteria, CA). The slides were mounted with cover slips and images were taken with a Leica DM5000 upright microscope (Leica Microsystems, Heidelberg, Germany). ImageJ 1.41 software was used to calculate the density of Hsp60-immunoreactive neurons in the DG and hippocampus proper (CA3, CA1) of Ctrls and MDA-stimulated rats. The densitometric analysis was performed on five fields per hippocampal sector, five sections per rat and in four rats per group. The objective lens used was 40×. The acquired images (RGB) were converted into grey scale images (32-bit) and inverted. Staining intensity of neurons was represented by a histogram and expressed as mean of pixel intensity (PI). The intensity of a pixel was expressed within a given range between a minimum (zero) and a maximum (255), where 0 corresponds to no positivity (black) and 255 to maximal positivity (in greyscale black and white, respectively).

Sections stained only with hematoxylin were run concurrently; pixel intensity was measured and subtracted from the Hsp60 quantification.

### Recruitment of patients

The study was carried out in accordance with the EU Council Directive for the use of human samples in research and approved by the local ethics committees at the IRCCS “NEUROMED” (Istituto Neurologico Mediterraneo), Pozzilli (IS), Italy. Patients were recruited at the Epilepsy surgery Unit of the IRCCS “NEUROMED”. All patients gave written informed consent to the collection of the blood samples and the processing of their personal data for clinical research purposes. We recruited 10 patients (Female/Male = 3:7; age: 23–49 years; mean age: 36.1 ± 9.18 years) affected by drug-resistant TLE evaluated for surgery by means of a non-invasive diagnostic protocol described in details elsewhere^55^. Whole blood samples were collected in EDTA-treated tubes on the day of admission into the Unit and at 30 minutes after the occurrence of a seizure. After a centrifugation at 2,000 × g for 10 min, plasma was collected, aliquoted, and stored at −80°C until use. Blood samples were collected from 10 age-matched controls (Female/Male = 3:7; age: 22–52 years; mean age: 36.3 ± 9.67 years) at the Unit of Internal Medicine of the *Azienda Ospedaliera Universitaria, Policlinico* Hospital, Palermo, Italy.

### ELISA tests

ELISA tests were performed as previously described^25^ using a commercial Hsp60 (human) enzyme immunoassay (EIA) kit (Enzo Life Sciences, Vinci, Italy) and a commercial EIA kit for Heat Shock 60 kD protein 1, chaperonin (HSPD1; rat) (Cloud-Clone Corp. Houston, TX). For the samples of human origin, the Hsp60 standard was diluted in standard diluent to generate a standard curve with six points, ranging from 3.125 to 100 ng/ml. For samples obtained from animals, Hsp60 standard was diluted in standard diluent to generate a standard curve with seven points, ranging from 3.12 to 200 ng/ml. Standard diluent alone was used as a 0 (zero) standard. Then, 100 μl of prepared standards and undiluted plasma was added in duplicate to wells of the immunoassay plate precoated with mouse monoclonal antibody specific for Hsp60 and incubated at 23°C for 1 h and at 37°C for 2 h for human and rat samples, respectively. After diluting the primary and secondary antibodies according to the manufacturer's instructions, 100 μl of anti-Hsp60 goat polyclonal antibody was added to each well and incubated at 23°C for 1 h and at 37°C for 1 h for human and rat samples, respectively. Subsequently, 100 μl of horse radish peroxidase conjugate anti-goat IgG was added to the plate and incubated at 23°C for 30 min and at 37°C for 30 min for human and rat samples, respectively, followed by 100 μl of 3,3′,5,5′-tetramethylbenzidine substrate for 15 min in the dark. Finally, 100 μl of Stop Solution was added, and absorbance was measured at 450 nm with a microplate photometric reader (DV990BV4, GDV, Milan, Italy). Sample concentration was calculated by interpolating the sample measurement in the standard curve. The sensitivity of the human Hsp60 EIA kit was determined to be 3.125 ng/ml. Human Hsp60 EIA kit is specific for Hsp60 and the Hsp60 ELISA has been certified for the detection of human Hsp60. The sensitivity of the rat Hsp60 EIA kit was determined to be 1.29 ng/ml and the assay has excellent specificity for detection of rat Hsp60. All experiments have been conducted according to the principles expressed in the last updated Declaration of Helsinki.

### Statistical analyses

Statistical analyses were performed using statistical software package GraphPad Prism4 (San Diego, CA). The data obtained were compared by the One-way ANOVA analysis of variance using Bonferroni post-hoc multiple comparisons. The data were expressed as means ± SD. Statistical significance was determined at the level of p ≤ 0.05. Pearson's correlation analysis was performed to describe relationships between the different parameters of MDA and the serum HSP60 levels measured at the end of the experiment (24 stimulations every 10 min). An alpha level of 0.05 was chosen as statistical threshold.

## Author Contributions

A.M.G. and G. Di Giovanni conceived the study and designed the experiments. A.M.G., G. Di Giovanni and A.J.L.M. wrote the manuscript. A.M.G., R.C., G.O. and M.P. performed experiments and analyzed data. G.D.G. and A.D. performed the recruitment of patients and the collection of blood samples. M.L.B. performed the animal tissue collection. M.V., R.M., F.B., C.P., A.B., G.Z., E.C. de M. and F.C. contributed to discussions. G. Di. Giovanni and F.C. provided funding. All authors reviewed the manuscript.

## Supplementary Material

Supplementary Information Supplementary information
