# Supplementary material for: Hsp60 response in experimental and human temporal lobe epilepsy
Source: Sci Rep. 2015 Mar 24;5:9434. doi: 10.1038/srep09434 (PMC4371150; doi:10.1038/srep09434)
Supplement: Supplementary Information [file srep09434-s1.pdf]

Hsp60 response in experimental and human temporal lobe epilepsy

Antonella Marino Gammazza, Roberto Colangeli, Gergely Orban, Massimo Pierucci, Giancarlo Di Gennaro, Margherita Lo Bello, Alfredo D’Aniello, Fabio Bucchieri, Cristoforo Pomara, Mario Valentino, Richard Muscat, Arcangelo Benigno, Giovanni Zummo, Everly Conway de Macario, Francesco Cappello, Giuseppe Di Giovanni, Alberto JL Macario

Supplementary figure S1

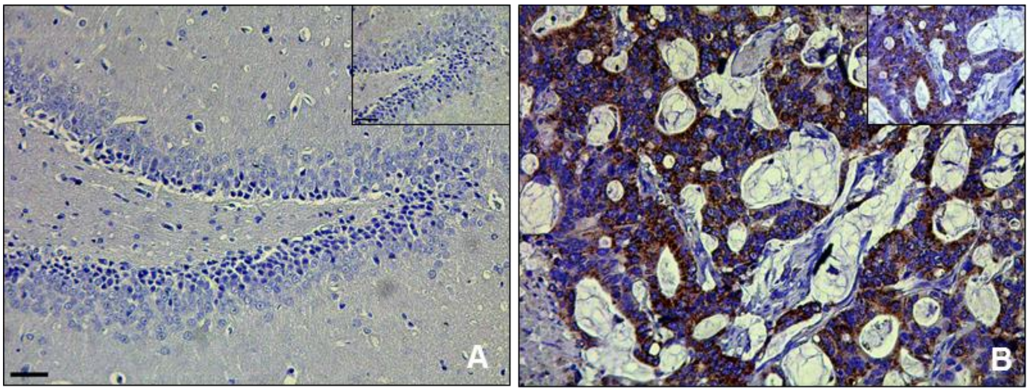

Figure S1: Positive and negative controls for immunohistochemistry. (A) Negative. Brain tissue was incubated with antibody diluent, without the primary antibody for Hsp60 and this was followed by incubation with secondary antibodies and detection reagents. Dentate gyrus (DG) is show with no reaction visible (B) Colorectal adenocarcinoma tissue was incubated with primary and secondary antibodies and with detection reagent. Typical positivity for Hsp60 is clearly visible in brown. Bar= 100 µm.
